# Redox mechanisms of cardiomyocyte mitochondrial protection

**DOI:** 10.3389/fphys.2015.00291

**Published:** 2015-10-26

**Authors:** Raquel R. Bartz, Hagir B. Suliman, Claude A. Piantadosi

**Affiliations:** ^1^Department of Anesthesiology, Duke University School of MedicineDurham, NC, USA; ^2^Department of Medicine, Duke University School of MedicineDurham, NC, USA; ^3^Department of Pathology, Duke University School of MedicineDurham, NC, USA; ^4^Durham Veterans Affairs HospitalDurham, NC, USA

**Keywords:** reactive oxygen species (ROS), mitochondria, heart, mitochondrial biogenesis, nitric oxide synthase, oxidative stress

## Abstract

Oxidative and nitrosative stress are primary contributors to the loss of myocardial tissue in insults ranging from ischemia/reperfusion injury from coronary artery disease and heart transplantation to sepsis-induced myocardial dysfunction and drug-induced myocardial damage. This cell damage caused by oxidative and nitrosative stress leads to mitochondrial protein, DNA, and lipid modifications, which inhibits energy production and contractile function, potentially leading to cell necrosis and/or apoptosis. However, cardiomyocytes have evolved an elegant set of redox-sensitive mechanisms that respond to and contain oxidative and nitrosative damage. These responses include the rapid induction of antioxidant enzymes, mitochondrial DNA repair mechanisms, selective mitochondrial autophagy (mitophagy), and mitochondrial biogenesis. Coordinated cytoplasmic to nuclear cell-signaling and mitochondrial transcriptional responses to the presence of elevated cytoplasmic oxidant production, e.g., H_2_O_2_, allows nuclear translocation of the Nfe2l2 transcription factor and up-regulation of downstream cytoprotective genes such as heme oxygenase-1 which generates physiologic signals, such as CO that up-regulates Nfe212 gene transcription. Simultaneously, a number of other DNA binding transcription factors are expressed and/or activated under redox control, such as Nuclear Respiratory Factor-1 (NRF-1), and lead to the induction of genes involved in both intracellular and mitochondria-specific repair mechanisms. The same insults, particularly those related to vascular stress and inflammation also produce elevated levels of nitric oxide, which also has mitochondrial protein thiol-protective functions and induces mitochondrial biogenesis through cyclic GMP-dependent and perhaps other pathways. This brief review provides an overview of these pathways and interconnected cardiac repair mechanisms.

Intact adult cardiomyocytes can be injured not only by ischemia, but also by various forms of oxidative and nitrosative stress following ischemia/reperfusion after myocardial infarction, sepsis-induced myocardial dysfunction and demand ischemia (Ferrari et al., 1989; Loeper et al., 1991a,b; Iqbal et al., 2002; Supinski et al., 2009). A major site of intracellular damage, particularly in the presence of pre-existing metabolic disease, such as diabetes, is the large population of mitochondria, which occupy 30% of the cardiomyocyte cytoplasmic volume (Laguens and Gómez-Dumm, 1967; Kane et al., 1975; Jennings and Ganote, 1976). However, the heart has an elegant system of anti-oxidant defenses and cell repair mechanisms that respond rapidly to and protect cardiomyocytes against oxidative and nitrosative stress, and which govern the maintenance and restoration of functional mitochondrial populations. This system involves the transcriptional relation of genes responsible for mitochondrial quality control (QC), an integrated process designed to optimize energy homeostasis. In cardiomyocytes, many of these mechanisms are redox-sensitive, and this short review concentrates on those mechanisms that are most important to the maintenance of cardiomyocyte function during periods of oxidative and nitrosative stress, and thereby serve to preserve cardiomyocyte viability and oppose apoptosis and necrosis.

## Production of reactive oxygen and nitrogen species

As highly metabolic cells, cardiomyocytes maintain a high cellular store of phosphocreatine and adenosine triphosphate (ATP), which is required for continuous cardiac function. The large-scale process of generating adenosine triphosphate (ATP) from carbon substrate, which in the heart relies mostly on fatty acids, also leads to the production of reactive oxygen and reactive nitrogen species (ROS/RNS) by the mitochondrial electron transport chain (ETC). This ROS production is primarily in the form of superoxide (^·^${\text{O}}_{2}^{-}$) and RNS in the form of peroxinitrite. Superoxide is generated by the incomplete one-electron reduction of oxygen mainly at Complexes I and III (Cadenas et al., 1977; Turrens et al., 1985; Aon et al., 2003; Chen et al., 2003; Murphy, 2009) and is highly reactive. Under normal mitochondrial conditions, ^·^${\text{O}}_{2}^{-}$ undergoes rapid dismutation either spontaneously or by mitochondrial (Mn) superoxide dismutase (SOD2) to hydrogen peroxide (H_2_O_2_). H_2_O_2_ exits the mitochondrion to the cytoplasm, where it is relatively soluble, and in the cytoplasm undergoes further catalysis to water (H_2_O) and oxygen (O_2_) by catalase (Cat), glutathione peroxidases, glutathione, thioredoxin, and the peroxiredoxins (Balaban et al., 2005; Aon et al., 2012). Additionally, thioredoxin reductase-2 has been shown to control thioredoxin-2 and peroxiredoxin-3 and thus controlling H_2_O_2_ emission from the mitochondria independent of glutathione reduction (Stanley et al., 2011). However, under certain circumstances, H_2_O_2_ in concert with endogenous production of carbon monoxide (CO) and nitric oxide (NO) serve as important redox signals for anti-oxidant protection and for the cellular repair mechanisms discussed in this review.

Tissue specific H_2_O_2_ production and its related signaling effects appear to be dependent on factors such as age, diet, and exercise capacity. For instance, elevated mitochondrial H_2_O_2_ is found in cardiac tissues of sedentary rats, and decreases with both exercise and high-fat, high sucrose diets unlike in skeletal muscle where a high-fat, high-sucrose diet leads to greatly elevated mitochondrial H_2_O_2_. These tissue-specific differences are due mainly to different levels of thioredoxin-2 reductase expression in cardiac compared to skeletal muscle in sedentary animals (Fisher-Wellman et al., 2013). Although redox-specific signaling capacities of different muscle types were not examined in this study, other studies have shown that when cellular ROS (H_2_O_2_) production rates are properly balanced by the presence of intracellular anti-oxidant enzymes like SOD2 and Cat, there is little or no oxidative stress and the intracellular homeostasis is maintained. However, when myocardial mitochondrial ROS generation exceeds the local antioxidant capacity, such as during ischemia/reperfusion and in sepsis (Ide et al., 1999; Gauthier et al., 2013; Cortassa et al., 2014), oxidative mitochondrial damage becomes problematic, and multiple intracellular adaptive mechanisms are up-regulated. These mechanisms result in the recruitment of cell pro-survival processes that afford tissue protection and prevent the progression to apoptosis and/or necrosis.

## Mitochondrial redox signaling of mitochondrial biogenesis

**Figure 1:**
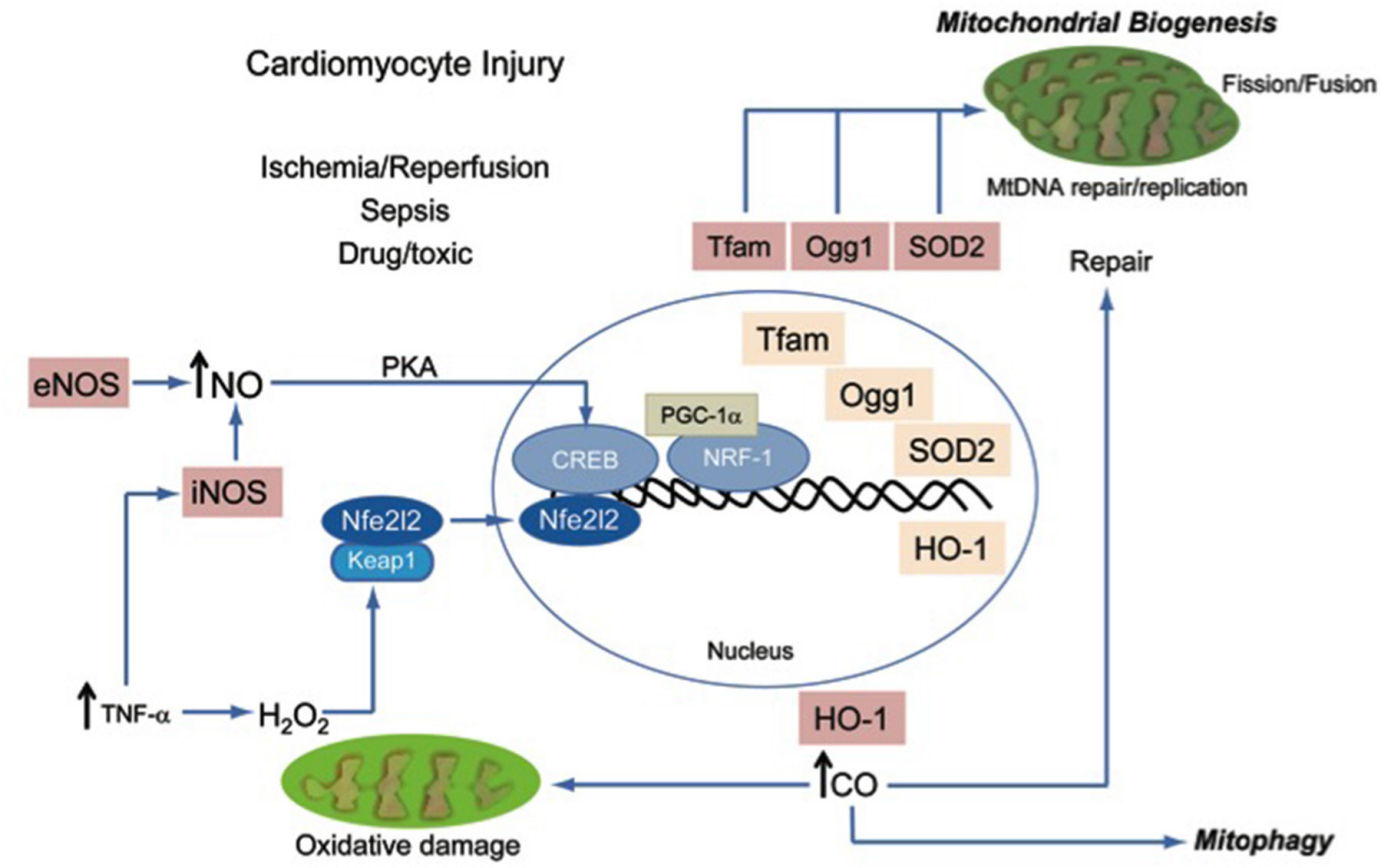
**A schematic diagram of known processes that are recruited to maintain mitochondrial quality control and to prevent energy failure during oxidative and nitrosative injury**.

One of the most important adaptive mechanisms in response to oxidative stress is genetic and involves the anti-oxidant response element (ARE) transcriptional pathway which responds to the presence of chemical electrophiles and to elevated cytoplasmic H_2_O_2_ content (Itoh et al., 1997). When cytoplasmic electrophilic or oxidative ([H_2_O_2_]) stress increases, the cytoplasmic protein and binding partner of Nuclear factor erythroid-derived-like 2 (Nfe2l2 or Nrf-2), Kelch-like ECH-associated protein 1 (Keap1), releases Nfe2l2, which translocates to the nucleus (Itoh et al., 1999b). In the nucleus, Nfe2l2 binds to ARE promoter regions of genes that carry an RTGACnnnGC motif including phase II detoxifying enzymes, certain anti-oxidant enzymes such as SOD2, cytoprotective enzymes such as heme oxygenase-1 (HO-1), and genes for signaling proteins required for mitochondrial biogenesis such as Nuclear Respiratory Factor-1 (NRF-1), and for mitochondrial DNA (mtDNA) repair such as 8-oxoguanine glycosylase (Ogg1), and several proteins discovered more recently that are required for mitophagy (Rushmore et al., 1991; Favreau and Pickett, 1995; Prestera et al., 1995; Alam et al., 1999; Itoh et al., 1999a,b; Jaloszynski et al., 2007; Cherry et al., 2014; Chang et al., 2015). Each of these proteins and related pathways function to protect cells from oxidative stress and to prevent apoptosis/cell death, especially via maintenance of mitochondrial biogenesis and mitophagy, which together comprise an integrated mitochondrial quality control (QC) system (Figure 1).

A metabolically active tissue, such as myocardium, may be particularly susceptible to ROS. For instance, the inflammatory cascade triggered by local TNF-α production as a result of myocardial infarction leads to mtDNA damage, to lipid peroxidation, and to protein oxidation (carbonylation). All of these oxidized products are potential stimuli for induction of cellular apoptosis (Irwin et al., 1999). In order to maintain adequate function in the face of this damage, the critical constituents of the mitochondria, such as mtDNA, proteins, and lipids must be repaired or replaced. However, massive damage cannot be repaired and the organelle must be removed by mitophagy. For instance, hyper-stimulation of the innate immune system, such as Toll-like receptor 4 by endotoxin and other microbial products released into the circulation leads to oxidative and nitrosative stress-induced cardiomyocyte dysfunction and subsequent mtDNA damage in the form of oxidized mtDNA lesions which must ultimately be removed or repaired in order for mtDNA replication and mitochondrial biogenesis to occur (Suliman et al., 2004). On the other hand, activation of mitophagy, a form of selective macroautophagy by which cells remove dysfunctional and ROS-producing mitochondria, isolates and contains these damage-causing organelles, and hence essentially functions as a macro antioxidant system. The proper ordering of these molecular repair mechanisms is necessary in order for mitochondrial biogenesis to ensue smoothly.

As mentioned, mitochondrial biogenesis requires the coordination of both the mitochondrial and the nuclear genomes and the synthesis, activation and nuclear translocation of several transcription factors and their nuclear co-activators including NRF-1, NRF-2 (mouse ortholog GABP-α), and PGC-1α, PGC-1β, and PRC (Evans and Scarpulla, 1990; Chau et al., 1992; Scarpulla, 1997, 2002; Wu et al., 1999; Andersson and Scarpulla, 2001; Lin et al., 2002; Kelly and Scarpulla, 2004; Gleyzer et al., 2005; Vercauteren et al., 2008). These nuclear transcription factors and their co-activators are responsible for the regulation of genes whose products regulate the hundreds of nuclear-encoded mitochondrial proteins (NEMPs) involved in the many functions of cardiac mitochondria including oxidative phosphorylation and calcium homeostasis. NEMPs also regulate the function of the mitochondrial genome (mtDNA). After synthesis, these proteins are imported into mitochondria by specialized outer and inner membrane protein complexes (Mokranjac and Neupert, 2009), also under the control of NRF-1.

The nuclear transcription factor NRF-1 and its co-activator, PGC-1α, indirectly regulate the mitochondrial genome by the up-regulation of mitochondrial transcription factor A (Tfam) and mitochondrial transcription factor B which enable transcription of the mitochondrial genome (Carter and Avadhani, 1994; Virbasius and Scarpulla, 1994; Dairaghi et al., 1995; Carrodeguas et al., 1996; Shadel and Clayton, 1996; Larsson et al., 1998; Falkenberg et al., 2002; Ekstrand et al., 2004; Gleyzer et al., 2005). Oxidative stress occurring in the myocardium as a result of ischemia/reperfusion injury or other cytotoxic insults, for example, in the form of cancer chemotherapeutics or in response to sepsis-induced oxidative stress leads to nuclear translocation of NRF-1 and up-regulation of its co-activator, PGC-1α (Suliman et al., 2004; Hickson-Bick et al., 2008). Transcription factor binding by one of the PGC-1α family members such as PGC-1β or PRC is required for mitochondrial biogenesis. In conjunction, adaptive cytoprotective mechanisms regulated by the endogenous gaseous signaling molecules are able to stimulate mitochondrial biogenesis.

## Physiologic carbon monoxide (CO) and mitochondrial biogenesis

A major example of such a cytoprotective pathway induced by oxidative and nitrosative stress involves the up-regulation of HO-1 (*Hmox1*), which catalyzes the breakdown of free heme, a cellular toxin. During periods of oxidative stress, HO-1 levels increase in the heart, and the enzyme produces physiologic carbon monoxide (CO) by breakage of the heme ring to form biliverdin (Maines, 1988; Maulik et al., 1996; Sharma et al., 1996). Although often known for its damaging properties to the cell, CO produced endogenously by HO-1 has been shown to offer multifaceted cytoprotection. For instance, CO, through the functional consequences of reduced (Fe^2+^) heme protein binding, induces targeted ROS signaling that initiates cellular repair mechanisms and mitochondrial biogenesis. The latter occurs in part through promotion of the cytoplasmic release and translocation of Nfe2l2 (Nrf-2) into the nucleus. As mentioned Nfe2l2 nuclear translocation leads not only to the induction of specific anti-oxidant enzymes, but also activation of NRF-1-dependent mitochondrial biogenesis (Suliman et al., 2007b; Piantadosi et al., 2008). Moreover, this redox-pathway is closely coupled to the mitophagy process that sequesters and eliminates damaged mitochondria from the cell (Strohecker and White, 2014; Chang et al., 2015). Current technological limits make cellular CO measurements difficult, particularly with respect to the spatial localization of CO production. Even though the local concentration at which CO is toxic is unknown, when circulating carboxyhemoglobin concentrations exceeds 15%, the decrease in hemoglobin oxygen carrying capacity is reduced enough to produce symptoms of CO poisoning.

The Nfe2l2 pathway for HO-1 induction and subsequent mitochondrial biogenesis are important for cytoprotection. It has been found using siRNA techniques that reduction of Nfe2l2 (Nrf-2) activity in Hep9C2 cardiomyoblasts results in reduced cell survival in response to hypoxia with or without reoxygenation (Kolamunne et al., 2013). In experimental models of congestive heart failure (CHF), decreased levels of certain cytochrome oxidase subunits, PGC-1α and the mitochondrial transcription factor, Tfam have been reported (Garnier et al., 2003). A cardiomyopathy induced in humans by a widely used chemotherapeutic agent, doxorubicin has been shown to directly damage mtDNA (Palmeira et al., 1997). Protection against doxorubicin-induced cardiomyopathy by HO-1 activation in the heart leads to the induction of mitochondrial biogenesis from the generation of physiological CO (Suliman et al., 2007a). Subsequent investigations have also shown that CO generated from HO-1 overexpression stimulates mitochondrial SOD-2 up-regulation and increases H_2_O_2_ production, which activates AKT/PKB, deactivates GSK3β and induces NRF-1 leading to the stimulation of mitochondrial biogenesis (Piantadosi et al., 2008). It has also been shown that the administration of a CO-releasing molecule leads to increased HO-1 expression levels and protects mice through restoration of cardiac mitochondrial biogenesis in response to sepsis. This suggests a mechanism by which the physiological stimulation of CO-dependent pro-survival mechanisms might have some future therapeutic potential (Lancel et al., 2009). In addition, transgenic mice overexpressing HO-1 is also protective against diabetic cardiomyopathy in mice, and this set of circumstances is associated with restored expression of Amp-associated kinase (AMPK) and AKT/PKB activation compared with mice with HO-1 deletion (Zhao et al., 2013). Such observations provide evidence of molecular linkage between the redox salvage pathways outlined here and the strictly energy-and/or calcium-dependent pathways of mitochondrial QC in the heart covered elsewhere (Calì et al., 2013; Dorn, 2015; Song et al., 2015).

A part of the mitochondrial QC program requires mitophagy—the uptake of dysfunctional mitochondria into lysozomes and their cellular degradation. An important role for Nfe2l2 (Nrf-2) and the HO-1/CO system has been shown in autophagic and presumably mitophagic pathways (Unuma et al., 2013a,b; Liu et al., 2014). Identification of damaged mitochondria and their packaging into autophagosomes requires the up-regulation and mitochondrial targeting of certain redox-sensitive proteins, most visibly p62 or Sequestosome-1 (Hamacher-Brady et al., 2006; Fujita et al., 2011; Huang et al., 2011; Stepkowski and Kruszewski, 2011; Darvekar et al., 2014; Haga et al., 2014; Chang et al., 2015). The p62 protein is an autophagy receptor that interacts directly with both the cargo to be degraded and the autophagy modifier protein, LC3. It is required for the formation and autophagic degradation of polyubiquitin-containing bodies, called ALIS (aggresome-like induced structures), serving to link ALIS to autophagosome formation (Fujita et al., 2011). This process maintains recycling of dysfunctional mitochondria within the cell.

## Nitric oxide (NO) induced signaling of mitochondrial biogenesis

Another major gaseous physiological mediator produced both constitutively and during ischemia/reperfusion and other types of cardiac damage is nitric oxide (NO). Although release of NO from ischemia may interact directly with ROS such as ^·^${\text{O}}_{2}^{-}$ to produce peroxinitrite (McBride et al., 1999; Paolocci et al., 2001), other evidence is beginning to emerge that NO plays a direct role in mitochondrial biogenesis as well as in mitochondrial QC in the heart. Using HL-1 cardiomyocytes, NO donors have been demonstrated to activate mitochondrial biogenesis (Vettor et al., 2014). Intracellular NO is produced by endothelial NO synthase (eNOS/NOS1), inducible NO synthase (iNOS/NOS2), or neuronal NO synthase (nNOS/NOS3). NO generated through NOS1 activity leads to the induction of mitochondrial biogenesis through guanylate cyclase activation and enhanced cyclic GMP and protein kinase A (PKA) activity (Nisoli et al., 2003, 2004; Signorile et al., 2014). Additionally, exercise-induced myocardial mitochondrial biogenesis is inhibited in mice with eNOS/NOS1 deletion (Vettor et al., 2014). And it has been shown that the renal hematopoietic hormone erythropoietin (EPO) induces mitochondrial biogenesis through EPO receptors in the mouse heart, and that this response is absent in mice lacking eNOS/NOS1 (Carraway et al., 2010).

The inducible NOS (NOS2 or iNOS) is also an important responder to oxidative stress and inflammation. NOS2 is induced by events that stimulate NF-κb nuclear translocation. Although not as well-studied within the heart as eNOS, in other organ systems the up-regulation of this enzyme isoform also promotes mitochondrial biogenesis (Suliman et al., 2010). Additionally, transgenic mice overexpressing NOS2 targeted to cardiomyocytes exhibit smaller infarct sizes in an ischemia/reperfusion model, and NOS2/iNOS overexpression has been found to inhibit the mitochondrial permeability transition, and to prevent ROS formation. However, it was not actually determined whether the mitochondrial biogenesis genetic program was up-regulated during the process (West et al., 2008).

Although beyond the scope of this review, it is important to note that controversy exists as to the presence of NOS within the mitochondria given NOS activity within the mitochondria has been previously reported (Giulivi et al., 1998; Ghafourifar et al., 1999). Most authors report an NOS1 (nNOS) variant (Holmqvist and Ekstrom, 1997; Kanai et al., 2001). Additionally, other authors have suggested localization of NOS2 to the mitochondrial by immunolabeling in addition to other organelles (Buchwalow et al., 1997) The role of so-called mtNOS is not clear and more work is needed to understand whether it has any role in mitochondrial biogenesis or mitochondrial QC in the heart.

Important to note, NO bioactivity plays a rather complex set of protective roles in the myocardium beyond those involved in mitochondrial QC. These include the ability of NO to form protein-thiol or SNO adducts in proteins in the form of protein nitrosylation (Stamler et al., 2001; Hogg et al., 2007; Broniowska and Hogg, 2012). These events in their early stages, such as the formation of SNO-proteins in mitochondria, are usually reversible, but rapidly can become chemically irreversible if allowed to persist or operate out of regulation (Anand and Stamler, 2012). The protective aspects appear to be related to the temporary inhibition of electron transport and to the regulation of ROS production by the organelles (Piantadosi, 2012). The extent to which this type of mechanism operates in the ischemic or failing heart and their impact on mitochondrial QC programming has not yet been fully explored, although recent descriptions have provided proof of principle of their biological importance (Ozawa et al., 2013; Sun et al., 2015).

In summary, the cytoprotective responses that have evolved to allow heart cells to respond, survive, and adapt to oxidative and nitrosative stress involve several novel, redox-based, signaling mechanisms. These are adaptive processes that occur generally before overt energy failure and result in heme oxygenase-1 up-regulation, nuclear translocation of Nfe2l2, induction of NRF-1 and its co-activator PGC-1α, the transcription of Tfam and its importation into mitochondria, the up-regulation of mtDNA repair enzymes and ultimately to mtDNA replication and to mitochondrial biogenesis. Both mitochondrial biogenesis and other organelle repair mechanisms occur in response to intracellular oxidative stress, but mitochondrial H_2_O_2_, production as well as endogenous enzymatic CO and NO production are able to induce the transcriptional program for mitochondrial biogenesis. This process along with the complementary and closely interrelated process of mitophagy protects and preserves a well-functioning population of mitochondria and allows the cell to reduce the ongoing contribution of damaged mitochondria to overall cellular oxidative stress. Together, these mechanisms are highly cardio-protective and preserve cardiomyocyte viability by limiting the activation of intrinsic apoptosis and by preventing energy failure and necrosis. A more thorough molecular understanding of the pathways that regulate and enhance mitochondrial QC has the potential to lead to novel therapeutic targets in a wide variety of heart diseases including ischemic, metabolic, and toxic cardiomyopathies.

## Author contributions

RB, HS, CP contributed equally to this design, draft, editing of the article.

## Funding

Grant support (RB: K08-GM087429 and CP: R01-AI095424, P01-HL08801) was provided by the National Institutes of Health.

### Conflict of interest statement

The authors declare that the research was conducted in the absence of any commercial or financial relationships that could be construed as a potential conflict of interest.

## Abbreviations

eNOS: endothelial nitric oxide synthase
iNOS: inducible nitric oxide synthase
NO: nitric oxide
TNF-α: tumor necrosis factor-α
H_2_O_2_: hydrogen peroxide
PKA: protein kinase A
Nfe2l2: nuclear factor (erythroid-derived 2)-like 2
Keap1: kelch-like ECH-associated protein 1
creb: cyclicAMP responsive element binding protein
PGC-1α: peroxisome proliferator-activated receptor gamma, coactivator 1α
NRF-1: nuclear respiratory factor-1
Tfam, transcription factor A: mitochondrial
OGG-1: 8-oxoguanine DNA glycosylase
SOD2: superoxide dismutase 2
HO-1: heme-oxygenase 1
CO: carbon monoxide
mtDNA: mitochondrial DNA.
